# Human Three-Finger Protein Lypd6 Is a Negative Modulator of the Cholinergic System in the Brain

**DOI:** 10.3389/fcell.2021.662227

**Published:** 2021-09-21

**Authors:** Dmitrii Kulbatskii, Zakhar Shenkarev, Maxim Bychkov, Eugene Loktyushov, Mikhail Shulepko, Sergey Koshelev, Igor Povarov, Alexander Popov, Steve Peigneur, Anton Chugunov, Sergey Kozlov, Irina Sharonova, Roman Efremov, Vladimir Skrebitsky, Jan Tytgat, Mikhail Kirpichnikov, Ekaterina Lyukmanova

**Affiliations:** ^1^Bioengineering Department, Shemyakin-Ovchinnikov Institute of Bioorganic Chemistry Russian Academy of Sciences (RAS), Moscow, Russia; ^2^Structural Biology Department, Shemyakin-Ovchinnikov Institute of Bioorganic Chemistry Russian Academy of Sciences (RAS), Moscow, Russia; ^3^Phystech School of Biological and Medical Physics, Moscow Institute of Physics and Technology, Moscow, Russia; ^4^Department of Molecular Neurobiology, Shemyakin-Ovchinnikov Institute of Bioorganic Chemistry Russian Academy of Sciences (RAS), Moscow, Russia; ^5^Brain Research Department, Research Center of Neurology, Moscow, Russia; ^6^Institute of Neuroscience, Nizhny Novgorod University, Nizhny Novgorod, Russia; ^7^Toxicology and Pharmacology, University of Leuven (KU Leuven), Leuven, Belgium; ^8^International Laboratory for Supercomputer Atomistic Modelling and Multi-Scale Analysis, National Research University Higher School of Economics, Moscow, Russia; ^9^Biological Faculty, Lomonosov Moscow State University, Moscow, Russia

**Keywords:** Lypd6, nAChR, cognitive function, synaptic plasticity, Ly6/uPAR, three-finger, Lynx1, Lypd6b

## Abstract

Lypd6 is a GPI-tethered protein from the Ly-6/uPAR family expressed in the brain. Lypd6 enhances the Wnt/β-catenin signaling, although its action on nicotinic acetylcholine receptors (nAChRs) have been also proposed. To investigate a cholinergic activity of Lypd6, we studied a recombinant water-soluble variant of the human protein (ws-Lypd6) containing isolated “three-finger” LU-domain. Experiments at different nAChR subtypes expressed in *Xenopus* oocytes revealed the negative allosteric modulatory activity of ws-Lypd6. Ws-Lypd6 inhibited ACh-evoked currents at α3β4- and α7-nAChRs with IC_50_ of ∼35 and 10 μM, respectively, and the maximal amplitude of inhibition of 30–50%. EC_50_ of ACh at α3β4-nAChRs (∼30 μM) was not changed in the presence of 35 μM ws-Lypd6, while the maximal amplitude of ACh-evoked current was reduced by ∼20%. Ws-Lypd6 did not elicit currents through nAChRs in the absence of ACh. Application of 1 μM ws-Lypd6 significantly inhibited (up to ∼28%) choline-evoked current at α7-nAChRs in rat hippocampal slices. Similar to snake neurotoxin α-bungarotoxin, ws-Lypd6 suppressed the long-term potentiation (LTP) in mouse hippocampal slices. Colocalization of endogenous GPI-tethered Lypd6 with α3β4- and α7-nAChRs was detected in primary cortical and hippocampal neurons. Ws-Lypd6 interaction with the extracellular domain of α7-nAChR was modeled using the ensemble protein-protein docking protocol. The interaction of all three Lypd6 loops (“fingers”) with the entrance to the orthosteric ligand-binding site and the loop C of the primary receptor subunit was predicted. The results obtained allow us to consider Lypd6 as the endogenous negative modulator involved in the regulation of the cholinergic system in the brain.

## Introduction

Nicotinic acetylcholine receptors (nAChRs) are ligand-gated ion channels and important participants of signaling in the nervous, endocrine, and immune systems of mammals (Wessler and Kirkpatrick, 2009). Dysfunction of the cholinergic signaling is associated with Alzheimer’s disease, Parkinson’s disease, some forms of cancer, and other pathologies (Schuller, 2009; Dineley et al., 2015).

The Ly-6/uPAR family consists of the proteins composed of the characteristic “three-finger” LU-domain(s) with a compact disulfide-stabilized β-structural core (“head”) and three protruding loops (“fingers”). The most known members of the Ly-6/uPAR family are snake α-neurotoxins and the receptor of urokinase-type plasminogen activator (uPAR), containing one and three LU-domains, respectively (Vasilyeva et al., 2017). There are also a lot of regulatory Ly-6/uPAR proteins containing the single LU-domain, and some of them are responsible for modulation of nAChRs in mammals (Ibañez-Tallon et al., 2002; Tekinay et al., 2009; Jensen et al., 2015; Arvaniti et al., 2016; Lyukmanova et al., 2016a,b). Regulatory Ly-6/uPAR proteins could be classified into two groups: secreted soluble proteins (e.g., SLURP-1 and SLURP-2) and GPI-anchored proteins (e.g., Lynx1, Lynx2, Lypd6, and Lypd6b). In the last case, the LU-domain is tethered to a cell membrane in the vicinity of the respective receptor (Loughner et al., 2016). For some of the Ly-6/uPAR proteins (e.g., Lynx1, PCSA, CD59, uPAR), both soluble and membrane-tethered forms have been reported (Fletcher et al., 1994; Thomsen et al., 2014; Jensen et al., 2015; Su et al., 2016).

Previous studies showed, that Lypd6 positively regulates the Wnt/β-catenin signaling in zebrafish and *Xenopus* embryos and in mammalian cells (Özhan et al., 2013). Lypd6 directly interacts with the Wnt coreceptor LRP6 (Zhao et al., 2018) and participates in embryogenesis supporting the patterning of the mesoderm and neuroectoderm during zebrafish gastrulation (Özhan et al., 2013). Expression of the *LYPD6* gene was demonstrated in various human tissues including heart, cerebral cortex, and spinal cord (Zhang et al., 2010), in the cerebral cortex and the spinal cord in mice (Darvas et al., 2009), and in the brain, lung, kidney, heart, liver, and prostate in rats (Arvaniti et al., 2016). The *Lypd6* gene is expressed in somatostatin-type GABA ergic interneurons in the deep layers of the adult mouse visual cortex (Demars and Morishita, 2014). GABAergic interneurons are implicated into the control of network oscillations, such as cortical theta-rhythms linked to spatial exploration, REM-sleep, memory, and information packaging (Colgin, 2013).

There are a number of data indicating that Lypd6 interacts with nAChRs and may be involved in the cholinergic signaling. Nicotine administration in the prenatal and early postnatal period results in the increased Lypd6 expression in the hippocampus of rats (Arvaniti et al., 2016). Neuronal *Lypd6* overexpression leads to increased locomotor activity and visceral hyperalgesia, typical for increased cholinergic tone, and to the enhancement of the nicotine-evoked calcium current amplitude in the trigeminal ganglion neurons of transgenic mice (Darvas et al., 2009). This calcium current can be inhibited by mecamylamine, but not by methyllycaconitine (MLA) or α-bungarotoxin (α-Bgtx), specific inhibitors of α7 type nAChRs (Darvas et al., 2009). From the other hand, knockout of the *Lypd6* gene significantly increases the amplitude of the nicotine-evoked current in the dorsal raphe nuclei in transgenic mice compared with WT mice (Arvaniti et al., 2018). In line with that, the water-soluble recombinant analog of Lypd6 fused to the *N*-terminal glutathione-S-transferase (GST-Lypd6) inhibits the nicotine-evoked current in the CA1 region of the hippocampus (Arvaniti et al., 2016). Affinity extraction from the human brain homogenate using magnetic beads coupled with GST-Lypd6 revealed that Lypd6 can interact with the α3, α4, α5, α6, α7, β2, and β4 subunits of nAChR (Arvaniti et al., 2016). So, despite the consensus that nAChRs are targets for Lypd6, the receptor specificity and direction of modulation are still debated, from the positive modulation of non-α7 receptors (Darvas et al., 2009) to the negative modulation of various receptor subtypes (Arvaniti et al., 2016, 2018).

In our studies of the structure and pharmacology of the Ly-6/uPAR proteins we used the recombinant isolated water-soluble LU-domains as an alternative strategy (Lyukmanova et al., 2011; Paramonov et al., 2020). The major advantage of this approach in comparison with the studies of membrane-bound GPI-tethered proteins (Ibañez-Tallon et al., 2002; Darvas et al., 2009; Ochoa et al., 2016) is an ability to control the protein concentration and to determine dose-response curve and other binding parameters. In our work, we also avoid the use of hybrid constructs like GST-Lypd6 (Arvaniti et al., 2016), since an additional protein or tag can alter the pharmacology of the Ly-6/uPAR protein (Chimienti et al., 2003; Lyukmanova et al., 2016a). Here, using the recombinant isolated water-soluble LU-domain of human Lypd6 (ws-Lypd6), we for the first time characterized the pharmacology of Lypd6 at different nAChR subtypes. We demonstrated that Lypd6 is the negative allosteric modulator of α3β4- and α7-nAChRs. Endogenous GPI-tethered Lypd6 colocalizes with α3β4- and α7-nAChRs in the primary cortical and hippocampal neurons, while ws-Lypd6 negatively modulates α7-nAChRs in rat hippocampal slices and suppresses the long-term potentiation (LTP) in mouse hippocampal slices. Thus, Lypd6 could play the role of the negative modulator in various brain processes associated with the function of α3β4- and α7-nAChRs, including cognition and memory. Comparison with the data obtained earlier for another Ly-6/uPAR protein ws-Lynx1 potentiating α7-nAChRs and LTP (Shenkarev et al., 2020), allows us to consider these two endogenous proteins as a pair of positive and negative modulators of the cholinergic system in the mammal brain.

## Materials and Methods

All animal care and experimental procedures were performed in accordance with the guidelines set forth by the European Communities Council Directive of November 24, 1986 (86/609/EEC) and were approved by the Ethical Committee of the Shemyakin-Ovchinnikov Institute of Bioorganic Chemistry RAS for the control of the maintenance and use of animals (protocol #222 from 13 February 2018).

### Production of Recombinant Proteins

The ws-Lypd6 and ws-Lypd6b proteins were produced and refolded from *E. coli* inclusion bodies as described previously (Paramonov et al., 2017). Protein purity, homogeneity, and correct folding were confirmed by HPLC, MALDI-MS, SDS-PAGE, and ^1^H-NMR spectroscopy. Protein concentration was quantified according to their molecular masses (MW_*ws*__–__*Lypd*__6_ = 10,928 Da, MW_*ws*__–__*Lypd*__6b_ = 10,546 Da) and the molar extinction coefficients (ε_280__,ws–Lypd6_ = 12,210 M^–1^⋅cm^–1^, ε_280__,ws–Lypd6b_ = 14,730 M^–1^⋅cm^–1^) by measuring the UV absorbance at 280 nm.

### Expression of Nicotinic Acetylcholine Receptors in *X. laevis* Oocytes

For expression of human nAChRs in *Xenopus* oocytes, the linearized plasmids containing the corresponded genes coding the receptor subunits (α1, α3, α4, α7, β2, β4, γ, δ, ε) were transcribed using the T7 or SP6 mMessage-mMachine transcription kit (Ambion^®^, Carlsbad, CA, United States). The harvesting of stage V–VI oocytes from anesthetized female *Xenopus laevis* frogs was previously described (Peigneur et al., 2019). Oocytes were injected with 50 nL of mRNA at a total concentration of 1 ng/nL using a micro-injector (Drummond Scientific^®^, Broomall, PA, United States). For heteromeric neuronal receptors a 1:1 ratio of α:β mRNA was used. For muscle receptors a 2:1:1:1 ratio of α:β:γ:δ/ε was used. The oocytes were incubated in a solution containing (in mM): 96 NaCl, 2 KCl, 1.8 CaCl_2_, 2 MgCl_2_, and 5 HEPES (pH 7.4), supplemented with 50 mg/L gentamycin sulfate.

### Electrophysiological Recordings in *X. laevis* Oocytes

Two-electrode voltage-clamp recordings were performed at room temperature (18–22°C) using a Geneclamp 500 amplifier (Molecular Devices^®^, Downingtown, PA, United States) controlled by a pClamp data acquisition system (Axon Instruments^®^, Union City, CA, United States). Whole-cell currents from oocytes were recorded 1–4 days after injection. Bath solution composition was (in mM): 96 NaCl, 2 KCl, 1.8 CaCl_2_, 2 MgCl_2_, and 5 HEPES (pH 7.4). Voltage and current electrodes were filled with 3 M KCl. Resistances of both electrodes were kept between 0.7 and 1.5 MΩ. During recordings, the oocytes were voltage-clamped at a holding potential of −70 mV and continuously superfused with solutions. Acetylcholine (ACh) was applied until the peak current amplitude was obtained, with 1–2 min washout periods between applications. nAChRs were gated by 100 ms pulses of ACh (100 μM for α1β1γδ, α1β1δε, α3β2, α3β4, α7; 10 μM for α4β2, and α4β4) at 2 mL/min. For the ACh dose-response curve at α3β4-nAChRs, the ACh concentration range was 10 nM–3 mM. Pre-incubation time of oocytes with ws-Lypd6 or ws-Lypd6b was 15 s for α7-nAChRs and 5 min for the other receptors. The solutions were prepared daily. The endogenous muscarinic receptors in oocytes were not blocked. Some oocytes expressing α7-nAChRs were tested with MLA as described in Shenkarev et al. (2020). MLA is a specific inhibitor of the α7 nicotinic receptors, but not of muscarinic ones. The almost complete inhibition of the ACh-evoked currents in the presence of 10 nM MLA indicates that currents associated with the activation of the muscarinic receptors by ACh are negligible, if any. Data were sampled at a frequency of 100 Hz and low-pass filtered at 20 Hz by using a four-pole Bessel filter. Peak current amplitude was measured prior to and following the incubation with ws-Lypd6 and ws-Lypd6b. Data were analyzed using pClamp Clampfit 10.0 (Molecular Devices^®^, Foster City, CA, United States) and Origin 7.5 software (Originlab^®^, Northampton, MA, United States).

### Primary Neuron Culture

The primary cultures of neurons from the cortex and hippocampus were obtained as previously described (Suntsova et al., 2013). Briefly, new-born rat pups were anesthetized, decapitated and the cortex or hippocampus were isolated, homogenized by scalpel and incubated 15 min in 0.8% trypsin solution in the DME medium. After that, the homogenate was centrifuged at 500 g for 2 min. Sediment was suspended in the Neurobasal-A medium (Gibco, United States) and dissociated by aspiration through a flame-polished 1 ml pipette repeated five times. Then, neurons were seeded on poly(L)-Lysine-coated glasses in 24-well plates, and the medium was changed after 1-h incubation in humidified atmosphere. To inhibit a growth of glial cells, 20 μM Cytarabin (Sigma-Aldrich) was added on the third day of cultivation. Neurons were cultivated for 12 days with a medium change every 4 days.

### Confocal Fluorescent Microscopy

Neurons from a primary culture were fixed in 4% paraformaldehyde for 1 h at 37°C, and sequentially incubated with the rabbit anti-Lypd6 (Antibodies Online, ABIN5582866, 1:1,000) and the mouse anti-α7-nAChR (Antibodies Online, Germany, ABIN5611363, 1:1,000) or mouse anti-α3-nAChR (Antibodies Online, ABIN5611357, 1:500) antibodies overnight and washed three times in PBS with 0.01% Tween-20. Second incubation was carried out with the goat anti-rabbit TRITC-labeled antibodies (Jackson Immunoresearch, 111-025-003, 1:500) and donkey anti-mouse AlexaFluor488-labeled antibodies (Jackson Immunoresearch, United Kingdom, 715-545-150, 1:1,000) for the Lypd6 and nAChRs visualization, respectively, during 1 h at room temperature. Cell nuclei were stained by Hoechst 33342. After washing three times in PBS with 0.01% Tween-20, the neurons were embedded in the Prolong Gold antifade mounting medium (Life Technologies, United States) and observed under x40 oil-immersion objective of the Carl Zeiss LSM710 inverted confocal laser scanning microscope (Carl Zeiss, Germany) using lasers with excitation of 405 nm (Hoechst 33342), 488 nm (Alexa-488), and 561 nm (TRITC) in line-by-line mode with alternating operation of the corresponding lasers and detection channels. The Pearson’s coefficient (Manders et al., 1993) was used for the analysis of the Lypd6 and nAChRs colocalization using the ZEN black 2.3 software (Carl Zeiss) with a manual threshold setup according to images stained separately by anti-Lypd6 or anti-nAChRs antibodies.

### Electrophysiological Recordings in Hippocampal Slices

Transverse hippocampal slices (350 μm thick) were prepared from juvenile Wistar rats (16–21-day-old). Prior to recording, the slices were incubated at 22°C for at least 1 h in artificial cerebrospinal fluid (ACSF) containing (in mM) 124 NaCl, 3 KCl, 2.4 CaCl_2_, 2.4 MgCl_2_, 26 NaHCO_3_, 1.25 NaH_2_PO_4_, 10 glucose, pH 7.4 and bubbled with 95% O_2_ and 5% CO_2_.

Whole-cell patch-clamp recordings were obtained from the interneurons in the hippocampal CA1 stratum radiatum using a microscope equipped with differential interference contrast optics (Axioskop FS, Zeiss AG, Germany). Recording patch electrodes (resistance of 5–7 MΩ) were filled with intracellular solution containing (in mM) 130 K-gluconate, 10 EGTA, 10 HEPES, 2 MgCl_2_, 1 CaCl_2_, 5 NaCl, pH 7.3. To diminish a desensitization of α7-nAChRs, choline-evoked currents were recorded at 22°C. The EPC-7 patch-clamp amplifier (HEKA, Germany) was used. The holding potential was −60 mV. Analog signals were lowpass filtered at 1–2 kHz, digitized at 50 kHz using the PCI-6281 interface (National Instruments, United States). WinWCP V5.2.7 software was used for the data acquisition and Clampfit 10.0 (Molecular Devices, Foster City, CA, United States) was used for the data analysis. Choline (1 mM) dissolved in ACSF was applied from borosilicate micropipettes (2–3 MΩ) via a pressure delivery system at a distance approximately 10–20 μm from the recorded soma. Pressure was maintained at 15 psi for 25 ms. Throughout the recording, the slices were perfused at a rate of ∼ 5 ml/min with bubbled ACSF. If needed, the aliquots of stock solutions of ws-Lypd6 (in 100% DMSO), α-bungarotoxin [α-Bgtx (Tocris), in water], or DhβE (Tocris, in water) were added to ACSF 20 min prior the recording of their effects. The final concentration of DMSO in ACSF did not exceed 0.1%.

### Long-Term Potentiation Recording in Hippocampal Slices

Transverse hippocampal slices (350 μm thick) were prepared from adult (4–6 month-old) male C57BL/6 mice. Prior to recording, the slices were incubated at 34°C for 1 h in ACSF containing (in mM) 124 NaCl, 3 KCl, 2.4 CaCl_2_, 1.3 MgCl_2_, 26 NaHCO_3_, 1.25 NaH_2_PO_4_, 10 glucose, pH 7.4 and bubbled with 95% O_2_ and 5% CO_2_. In some experiments, ACSF additionally contained 1 μM of ws-Lypd6 or 10 nM of α-Bgtx.

Field excitatory postsynaptic potentials (fEPSPs) were recorded under a visual guidance of microscope equipped with infrared differential interference contrast optics (Olympus BX51WI). The field potentials were recorded and filtered using the Multiclamp 700B amplifier (Axon Instruments, United States) and PCI-6281 interface. The stimulation was performed using the Digitimer DS3 constant current stimulator. WinWCP V5.2.7 software was used for data acquisition. The data were processed using Clampfit 10.0 (Molecular Devices, United States).

The ACSF-filled recording electrode (1–3 MΩ) was positioned within the hippocampal CA1 stratum radiatum. Synaptic responses were evoked by a paired-pulse stimulation of the Schaffer collaterals area with a bipolar electrode. Stimulus intensity was adjusted to elicit 40% of maximal fEPSP amplitude. The 50 ms interpulse interval was used, the stimulations were repeated with the 20 s interval. After 20 min baseline recording, LTP was induced by the high frequency stimulation (HFS) protocol: four 100-pulse trains at 100 Hz, 5 min apart. fEPSPs were then recorded for 60 min. If needed, 1 μM of ws-Lypd6 or 10 nM of α-Bgtx were added in ACSF 1 h prior the recordings and were presented in ACSF during the entire recording time. Slope of the post-tetanic response was normalized to the averaged slope of the baseline responses.

### Computational Modeling of a7-nAchR/Lypd6 Interaction

Homology model of the extracellular ligand-binding domain of α7-nAChR (α7-ECD) was constructed using the crystal structure of the α7/AChBP chimera as a template (PDB Id 3SQ9; Li et al., 2011) and the MODELLER 8.2 software (Martí-Renom et al., 2000). The complex was built using customized ensemble protein–protein docking procedure (Supplementary Figure 2) subdivided into several steps similarly to our previous works (Lyukmanova et al., 2015, 2016b). Briefly:

1. Molecular dynamics (MD) of the α7-ECD model in a water box was calculated using GROMACS 5.1.2 (Abraham et al., 2015) with Gromos96 45a3 parameters set and the SPC water model. Two independent 200-ns MD trajectories were calculated and combined. Conformational clustering was performed using the united 400-ns MD trajectory with the Gromos clustering algorithm and a distance cut-off of 0.25 nm. The intersubunit interfaces demonstrated a different position of the loop C of the primary (+) subunit. Clustering of various subunit interfaces yielded in 9 and 11 structures with a “closed” and “open” orthosteric ligand-biding pocket, respectively (Lyukmanova et al., 2016b).

2. For ws-Lypd6 in water, six MD trajectories of 200 ns each were calculated, starting with the most dissimilar NMR structures (PDB Id 6IB6; Paramonov et al., 2020): three trajectories for ws-Lypd6 and three trajectories for the protein with removed *C*-terminal “tail” (Leu83-Ala95). Conformational clustering was performed based on the united 600-ns MD trajectory for the protein without the *C*-terminal “tail.” Clustering with a distance cut-off of 0.25 nm resulted in the conformational ensemble containing 23 structures of ws-Lypd6.

3. Protein–protein docking was performed for the α7-ECD ligand-binding site/ws-Lypd6 system with the ZDOCK software (Chen et al., 2003). For each pair of the α7 ligand-biding site and ws-Lypd6 structures, two protein–protein docking runs were carried out. In the first run, we restricted the interaction of the Lypd6 “frontal” half (containing three “fingers” and predicted binding-site #1) with the receptor’s orthosteric binding site, using standard ZDOCK “block” option. In the second run, we restricted the interaction of the Lypd6 “head” (containing predicted binding-site #2) with the same receptor site. In total, 2 × 9 × 23 = 414 and 2 × 11 × 23 = 506 docking runs (“closed” and “open” α7-ECD ligand-biding pocket, respectively) were made. For each run, ZDOCK systematically generated 2000 models of the complex; 100 top-scoring structures were used for the further analysis.

4. The obtained 92,000 docking solutions were “post-filtered” using our in-house re-scoring protocol that requires: (a) ws-Lypd6 has significant contact area with the receptor (>4 nm^2^); (b) number of “good” contacts (h-bonds, ionic bridges and specific stacking) is above 14, and (c) complementarity of hydrophobic/hydrophilic properties in the complex is > 0.65. Analysis of these properties in the complexes was done with the PLATINUM software (Pyrkov et al., 2009). The post-filter resulted in the 39 “good” solutions (“fingers”/”closed” — 4; “fingers”/”open” — 20; “head”/“closed” — 6; “head”/“open” — 9).

5. The resulting “good” solutions were further filtered using specific requirements for the position of the receptor binding site and the *N*- and *C*-termini of the ws-Lypd6 protein. It was required that: (a) Lys8 or Arg17 of ws-Lypd6 (predicted binding-site #1), or Arg26 or Arg29 of ws-Lypd6 (predicted binding-site #2) participate in the ionic interactions with negatively charged residue(s) of α7-ECD; (b) ws-Lypd6 forms “favourable” contacts with the C loop of the primary receptor subunit; (c) *C*-terminus of ws-Lypd6 has realistic position relative to the expected membrane surface (native Lypd6 is tethered to the membrane by the GPI-anchor connected to the *C*-terminus via a relatively long 19-residue linker); (d) *N*-terminus of ws-Lypd6 has no direct contacts with α7-ECD (native Lypd6 has an additional *N*-terminal 25-residue sequence). The second post-filter resulted in the three solutions (“fingers”/“closed”—1; “fingers”/“open”—1; “head”/“open”—1). The topology of α7-ECD/ws-Lypd6 interactions in the first two solutions was very similar. Thus, our ensemble docking protocol resulted in only two models.

### Statistical Analysis and Curve Fitting

Data are presented as mean ± SEM. Sample numbers (n) are indicated in the figure legends and figures. Statistical analysis was done using two-tailed *t*-test or one sample two-tailed *t*-test as indicated in the figure legends. Differences in the groups were considered statistically significant at *p* < 0.05. To assess the concentration-response relationships, the normalized data points were fitted with the Hill equation: y(%) = 100%–A_0_(%)/[1 + (EC_50_/[protein])^*nH*^], where y(%) is the amplitude of the protein-induced effect, A_0_(%) is the maximal amplitude of the protein-induced effect, EC_50_ is the protein concentration at half-maximal efficacy, [protein] is the protein concentration, and nH is the Hill coefficient. Analysis was performed using the GraphPad Prism 6.0 software.

## Results

### Lypd6 Inhibits ACh-evoked Currents at α3β4- and α7-nAChRs Expressed in *X. laevis* Oocytes

**FIGURE 1:**
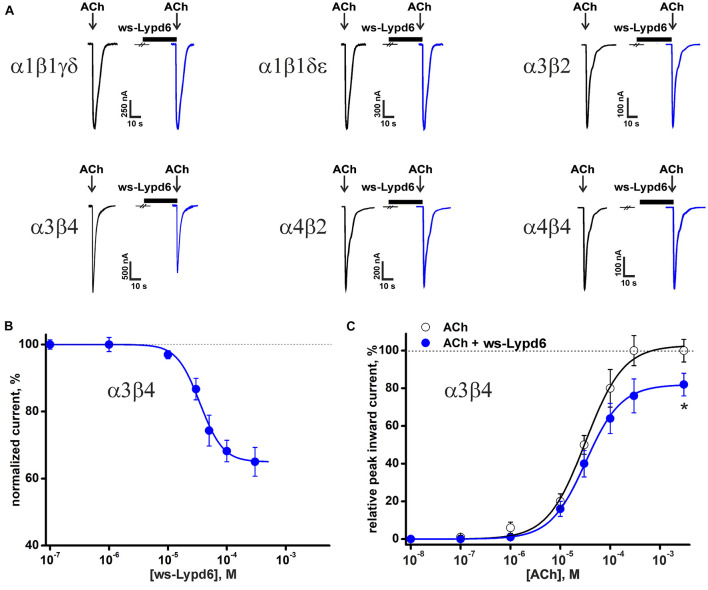
Effect of ws-Lypd6 on heteromeric nAChRs expressed in *X. laevis* oocytes. **(A)** Representative responses to 100 ms pulses of ACh (100 μM for α1β1γδ, α1β1δε, α3β2, α3β4; 10 μM for α4β2, and α4β4) recorded in the presence or absence of 30 μM ws-Lypd6. Pre-incubation time of oocytes with ws-Lypd6 was 5 min. **(B)** Dose-response curve for ws-Lypd6 inhibition of ACh-evoked currents at α3β4-nAChRs. The data are normalized to the peak amplitude of current recorded without ws-Lypd6 (100%) and presented as mean ± SEM (*n* = 6 oocytes). The data were fitted by the Hill’s equation resulted in IC_50_ for ws-Lypd6 of 34.9 ± 1.2 μM, A_0_ of 33 ± 5%. **(C)** Dose-response curves of ACh at α3β4-nAChRs in absence (open symbols) and presence of 35 μM ws-Lypd6 (closed symbols). The peak inward current amplitudes were normalized to the peak current obtained by the application of 1 mM ACh and plotted against the different concentrations of ACh applied in control. The concentration-response curve in presence of ws-Lypd6 was normalized to the control. The obtained EC_50_ values for ACh are 29.8 ± 4.7 μM and 31.3 ± 6.2 μM in absence and presence of ws-Lypd6, respectively. * (*p* < 0.05) indicates significant difference from the control value (100%, max. response for ACh without ws-Lypd6) (one sample *t*-test).

**FIGURE 2:**
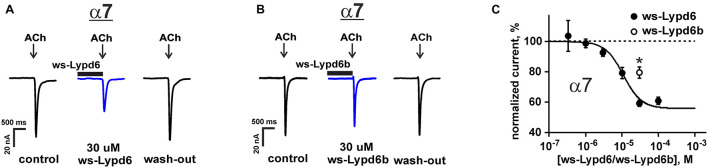
Ws-Lypd6 inhibits homomeric α7-nAChR expressed in *X. laevis* oocytes. **(A,B)** Representative responses to 100 ms pulses of 100 μM ACh recorded in the presence or absence of 30 μM ws-Lypd6 or ws-Lypd6b. Pre-incubation time of oocytes with ws-Lypd6 or ws-Lypd6b was 15 s. **(C)** Dose-response curve for the ws-Lypd6 inhibition of ACh-evoked currents at α7-nAChRs (closed symbols). The data are normalized to the peak amplitude of current recorded without ws-Lypd6 and ws-Lypd6b (100%) and presented as mean ± SEM (*n* = 8 oocytes from 3 animals). The data were fitted by the Hill equation resulted in IC_50_ for Lypd6 of 10.9 ± 1.8 μM, nH of 1.6 ± 0.4, and A_0_ of 46 ± 4%. Response to 30 μM ws-Lypd6b is shown by open symbol (*n* = 4 oocytes from 2 animals). * (*p* < 0.05) indicates significant difference of ws-Lypd6 and ws-Lypd6b effects (*t*-test).

The previously reported binding of GST-Lypd6 to the different nAChR subunits in the human brain homogenate (Arvaniti et al., 2016) implies that various nAChR subtypes may be targeted by Lypd6. To investigate the receptor specificity, we studied the effect of 1 and 30 μM ws-Lypd6 on a set of nAChRs (α1β1γδ, α1β1δε, α3β2, α3β4, α4β2, α4β4, and α7) expressed in *X. laevis* oocytes using two-electrode voltage clamp. We did not observe a potentiation of any of nAChRs studied, while the significant inhibition of the ACh-evoked current amplitude by 30 μM ws-Lypd6 was observed at α3β4- and α7-nAChRs (Figures 1A, 2A). The inhibition of the ACh-evoked responses at α3β4- and α7-nAChRs by ws-Lypd6 was concentration-dependent and characterized by the IC_50_ values of 34.9 ± 1.2 μM and 10.9 ± 1.8 μM and maximal reduction of the current amplitude of ∼ 35 and 46% relative to the control, respectively (Figures 1B, 2C). The response of the same oocyte to ACh in the absence of ws-Lypd6 was used as the control for each data point with the ws-Lypd6 pre-incubation. Notably, the application of ws-Lypd6 alone did not elicit currents at the receptors, and the observed inhibition was completely reversible (Figure 2A).

To study a possible competition between ws-Lypd6 and the agonist ACh for the interaction with the nicotinic receptors, we measured the concentration-response curves for ACh at α3β4-nAChRs in the absence and presence of 35 μM ws-Lypd6. The application of ws-Lypd6 did not significantly affect the EC_50_ value of ACh (changed from 29.8 ± 4.7 to 31.3 ± 6.2 μM), although the significant decrease of the maximal amplitude of the ACh-evoked current (from 100 to ∼82%) was observed (Figure 1C). Thus, ws-Lypd6 does not compete with ACh for the binding with α3β4-nAChRs. In addition, we did not observe any changes in the shape of the ACh responses at α3β4- and α7-nAChRs under the application of ws-Lypd6. Thus, the protein does not significantly change the properties of the desensitization process. All together obtained data permit us to classify ws-Lypd6 as the negative allosteric modulator of α3β4- and α7-nAChRs. Observed decrease in the agonist-evoked response amplitude without the significant effect on the response decay rate indicates that ws-Lypd6 hampers a transition from the resting to open channel state upon the activation by the agonist. Such ligand can be referred to as the type I allosteric modulator (Faghih et al., 2008).

The inhibitory effect of the homologous Ly-6/uPAR protein ws-Lypd6b, having the 54% similarity of the amino acid sequence with ws-Lypd6 (Supplementary Figure 1), was studied at α7-nAChRs using a single 30 μM concentration (Figures 2B,C). As compared with ws-Lypd6, the application of ws-Lypd6b resulted in the significantly lower inhibition of the ACh-evoked currents (reduction of the current amplitude by ∼40 and 20% for ws-Lypd6 and ws-Lypd6b, respectively, Figure 2C).

### Lypd6 Colocalizes in the Cortical and Hippocampal Neurons With Nicotinic Acetylcholine Receptors Containing the α3 and α7 Subunits

**FIGURE 3:**
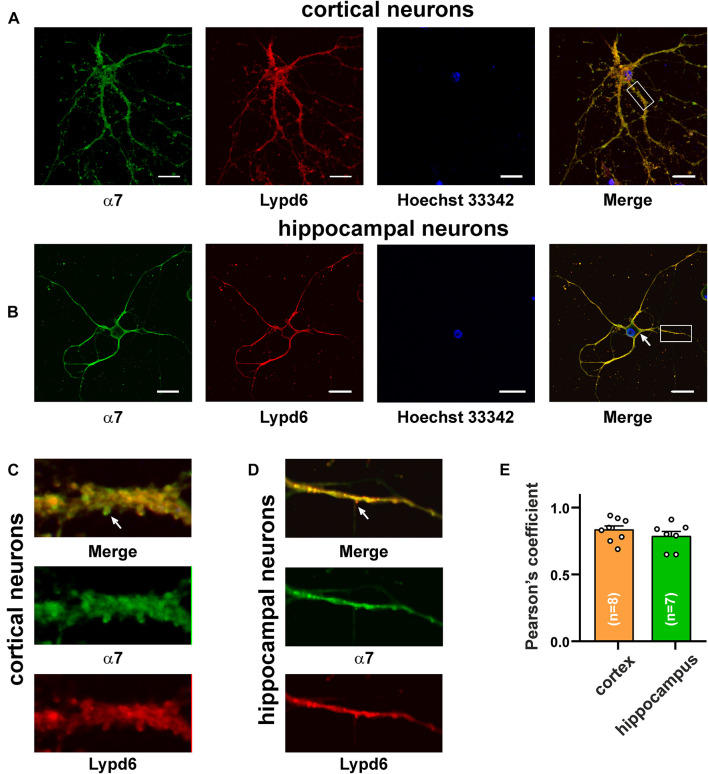
Co-localization of endogenous Lypd6 and nAChRs containing α7 subunit in primary cortical and hippocampal neurons. Cells were sequentially incubated with the mouse anti-α7/rabbit anti-Lypd6 antibodies and secondary anti-mouse Alexa-488 (green)/anti-rabbit TRITC (red) labeled antibodies, respectively. **(A,C)** Colocalization of Lypd6 and the α7 subunit in the cortical neurons. **(B,D)** Colocalization of Lypd6 and the α7 subunit in the hippocampal neurons. Scale bar 10 μm. **(C,D)** Enlarged fragments showed by white rectangles on **(A,B)**, respectively. Points with the different Lypd6 and α7 expression are shown by arrows. **(E)** Pearson’s correlation coefficients of the Lypd6 and α7 colocalization in primary cortical and hippocampal neurons (*n* = 4, microphotographs).

**FIGURE 4:**
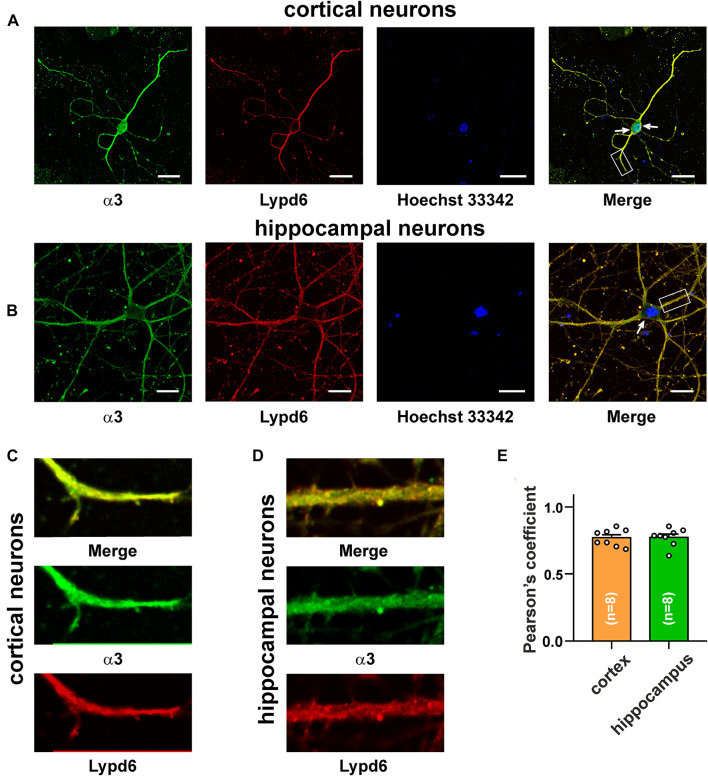
Co-localization of endogenous Lypd6 and nAChRs containing α3 subunit in primary cortical and hippocampal neurons. Cells were sequentially incubated with the mouse anti-α3/rabbit anti-Lypd6 antibodies and secondary anti-mouse Alexa-488 (green)/anti-rabbit TRITC (red) labeled antibodies, respectively. **(A,C)** Colocalization of Lypd6 and the α3 subunit in the cortical neurons. **(B,D)** Colocalization of Lypd6 and the α3 subunit in hippocampal neurons. Scale bar 10 μm. **(C,D)** Correspond to the enlarged fragments showed by white rectangles on **(A,B)**, respectively. Points with the different Lypd6 and α3 expression are shown by arrows. **(E)** Pearson’s correlation coefficients of the Lypd6 and α3 colocalization in primary cortical and hippocampal neurons (*n* = 4, microphotographs).

The main mammalian brain areas important for learning and memory are the cortex and hippocampus (Taly et al., 2009). *Lypd6* mRNA was found both in the murine cortex and hippocampus (Darvas et al., 2009), but little is known about the Lypd6 expression in the neurons at a protein level. α7-nAChR is the most widespread nicotinic receptor in the mammalian brain, and particularly in the hippocampus (Taly et al., 2009). To investigate a possible expression of Lypd6 in the neurons and its colocalization with α3β4- and α7-nAChRs, the primary cortical and hippocampal rat neurons were stained by the antibodies against Lypd6 and the α7 and α3 nAChR subunits (Figures 3, 4). Expression of the Lypd6 protein was observed in the soma and dendrites of the neurons, and the almost complete colocalization with nAChRs containing the α7 subunit was revealed (Figure 3, the Pearson’s correlation coefficients were 0.83 ± 0.03 and 0.78 ± 0.03 for the cortex and hippocampal neurons, respectively). However, the distribution of Lypd6 and α7-nAChRs were not uniform, and microphotographs demonstrated some points where larger amounts of Lypd6 as compared to α7-nAChRs (and vice versa) were presented (Figures 3C,D, arrows). In the case of the α3 subunit containing nAChRs, the similar degree and uniformity of colocalization with Lypd6 was observed in the cortical and hippocampal neurons (Figure 4, the Pearson’s correlation coefficients were 0.78 ± 0.02 in the both cases).

### Lypd6 Inhibits Choline-Evoked Current at α7-nAChRs in the Hippocampus

**FIGURE 5:**
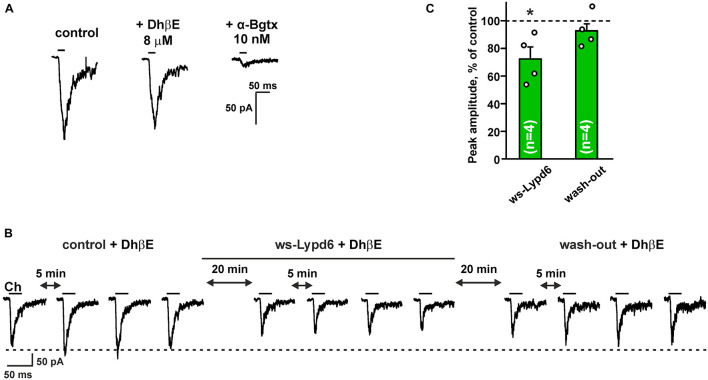
Ws-Lypd6 inhibits choline-evoked currents at nAChRs in the hippocampus. **(A)** Representative responses to 1 mM choline pulses in the absence and presence of 8 μM DhβE or 10 nM α-Bgtx. **(B)** Representative responses to 25 ms pulses of 1 mM choline (Ch) recorded in one cell in the absence and presence of 1 μM ws-Lypd6. **(C)** Peak amplitude of the choline-evoked currents in the presence of 1 μM ws-Lypd6 and after wash-out recorded in four different cells taken from different animals (*n* = 4 slices, four rats). Four responses (like shown in **B**) were averaged for each slice and normalized to the average control amplitude (100%, shown by dotted line). The data are presented as mean ± SEM. * (*p* < 0.05) indicates significant difference from the control value (*t*-test). Experiments in **(B,C)** were performed in presence of 8 μM DhβE.

Hippocampus is the brain region that is important for the formation and storage of episodic and semantic declarative memories (Avchalumov and Mandyam, 2021). CA1 str. radiatum of the hippocampus mainly contains the α4β2- and α7- nicotinic receptors (Taly et al., 2009). To further investigate the functional role of Lypd6 in the central nervous system, we tested an activity of ws-Lypd6 at native nAChRs in rat hippocampal slices. Application of choline induced the robust currents in the interneurons of CA1 str. radiatum (Figure 5A). Usage of 8 μM DhβE or 10 nM α-Bgtx (selective inhibitors of α4β2- and α7-nAChRs, respectively) revealed the significant inhibition of the choline-evoked currents only by α-Bgtx (Figure 5A). Thus, the observed currents were mainly due to the activation of α7-nAChRs. Nevertheless, to suppress the possible currents through α4β2-nAChRs, 8 μM DhβE was applied to ACSF in all subsequent experiments. The application of 1 μM ws-Lypd6 to ACSF significantly reduced the amplitude of the choline-evoked currents up to ∼72% of the control value (Figures 5B,C). Washout by ACSF returned the amplitude of the choline-evoked currents to the control level (Figures 5B,C), confirming that the binding of ws-Lypd6 to α7-nAChRs is reversible.

### Lypd6 Suppresses Long-Term Potentiation in the Hippocampus

**FIGURE 6:**
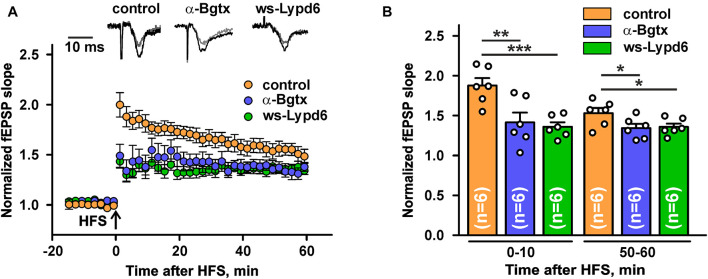
Ws-Lypd6 suppresses long-term potentiation (LTP) in the mice hippocampal slices. **(A)** Averaged 1 h LTP responses in hippocampal slices after 1 h preincubation in ACSF containing 1 μM ws-Lypd6 or 10 nM α-Bgtx (in each case *n* = 6 slices, six mice). The same compounds were present in ACSF during recording. Representative normalized fEPSP traces are shown above: gray—baseline, black—post-tetanic recording. **(B)** Normalized fEPSP slopes averaged over 0–10 and 50–60 min after HFS. The data are presented as mean ± SEM. * (*p* < 0.05), ** (*p* < 0.01), and *** (*p* < 0.001) indicate significant difference (*t-*test).

Nicotinic receptors containing the α7 subunit are involved in the glutamate release and synaptic plasticity (Lozada et al., 2012; Koukouli and Maskos, 2015; Bali et al., 2017; Nicholson and Kullmann, 2021). To study a possible ws-Lypd6 effect on the synaptic plasticity, we performed LTP recordings in hippocampal slices of C57BL/6 mice. Incubation of hippocampal slices in ACSF containing 1 μM ws-Lypd6 significantly suppressed LTP (decrease in the slope of fEPSP) during all 60 min of the recording after HFS as compared to the control (Figure 6). Similar effect was observed upon incubation of the slices with 10 nM α-Bgtx. These data, together with the data on the inhibition of α7-nAChRs in hippocampal slices by both α-Bgtx and ws-Lypd6 (Figure 5), indicate the involvement of the same target in the LTP suppression by the snake toxin and human neuromodulator.

### Analysis of the ws-Lypd6 Surface Revealed Possible Nicotinic Acetylcholine Receptor Binding Interface(s)

**FIGURE 7:**
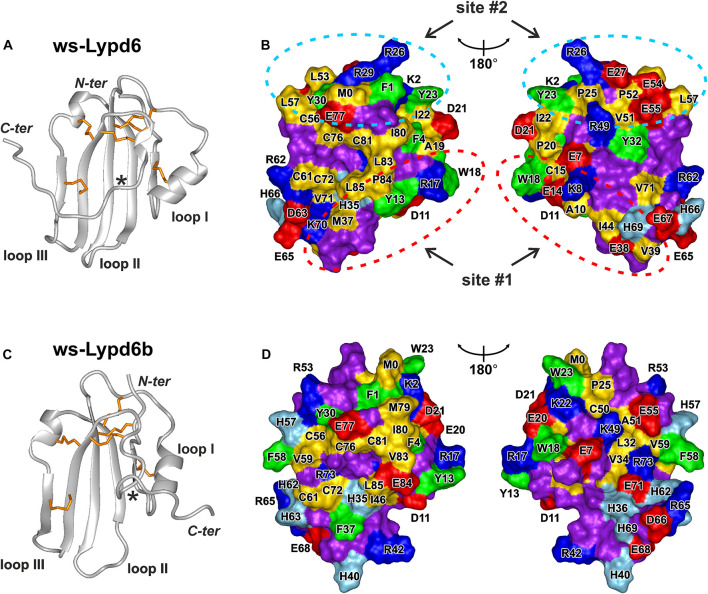
Spatial structures and molecular surfaces of ws-Lypd6 **(A,B)** and Lypd6b **(C,D)**. **(A,C)** The disulfide bonds are in orange. Only one conformation of the flexible *C*-terminal “tail” is shown for each protein. The *C*-termini of the structured LU-domains (Leu85) are marked with asterisks. **(B,D)** Two-sided view of the molecular surfaces. Only LU-domains without *C*-tails (terminated at Leu85) are shown. The hydrophobic (Ala, Met, Ile, Leu, Val, Cys, and Pro), aromatic (Phe, Trp, Tyr), polar (Asn, Gln, Gly, Ser, and Thr), positively charged (Arg and Lys), negatively charged (Asp and Glu), and His residues are colored in yellow, green, violet, blue, red, and cyan, respectively. Two possible receptor-binding sites responsible for the Lypd6 interaction with nAChRs are highlighted by dashed ellipses. Orientation of the molecular surfaces in the left parts of the panels **(B,D)** is identical to the molecules orientation in **(A,C)** PDB codes: ws-Lypd6—6IB6, ws-Lypd6B—6ZSO (Paramonov et al., 2020).

The previously observed competition of GST-Lypd6 with α-Bgtx for the binding to the α7 nAChR subunit (Arvaniti et al., 2016) implies that Lypd6 binds to the receptor near the orthosteric ligand-binding site. In all nAChR subtypes this site is located at the interface between the primary (+) and complementary (−) subunits (Huang et al., 2013; Noviello et al., 2021). The binding pocket for orthosteric ligands is covered by the loop C of the primary receptor’s subunit, and the bound ligands usually contact this loop (Hansen et al., 2005). The loop C has overall negative charge and the ligand-binding site contains a large proportion of aromatic residues (Grutter and Changeux, 2001; Li et al., 2011). Therefore, the active sites of the peptide ligands interacting with nAChRs (e.g., three-finger snake toxins) frequently are formed by the positively charged (Arg, Lys) and aromatic (Phe, Trp, Tyr) residues (Lyukmanova et al., 2016c). Based on this empirical rule, we analyzed the surface of the ws-Lypd6 molecule, which structure (Figures 7A,B) recently was determined by NMR (Paramonov et al., 2020; PDB id 6IB6). Two possible receptor-binding sites were identified in the LU-domain of Lypd6: site #1 in the loop I, where the charged side-chains of the Lys8 and Arg17 residues are located in close proximity to the aromatic groups of the Tyr13 and Trp18 residues (Figure 7B, red ellipses), and site #2 in the “head” region, where the side-chains of the Arg26, Arg29, Phe1, and Tyr23 residues protrude from the protein core (Figure 7B, blue ellipses).

### *In silico* Modeling of the Interaction Between ws-Lypd6 and α7-nAChR

To assess a probable mode of the interaction between ws-Lypd6 and its target in the hippocampus, where the negative modulation of α7-nAChRs and LTP by ws-Lypd6 was found (Figures 5, 6), we modeled the complex of ws-Lypd6 with the extracellular domain of α7-nAChR (α7-ECD). Besides the structurally conserved LU-domain, the native Lypd6 protein contains the relatively long *N*- and *C*-terminal sequences and *C*-terminally attached GPI-anchor (Supplementary Figure 1). The *N*-terminal 25-residue sequence prevents the correct folding of recombinant ws-Lypd6 (Paramonov et al., 2017) and, according to the predictions of the JPred4 algorithm (Drozdetskiy et al., 2015), does not form elements of the secondary structure (α-helices or β-sheets). At the same time, according to the previously obtained NMR data, the *C*-terminal sequence is disordered and has no specific structure in solution (Paramonov et al., 2020). Therefore, in this study, we assumed that the flexible *N*- and *C*-terminal regions of Lypd6 are not involved in the binding to nAChRs. To model the interaction with α7-ECD, we used the isolated LU-domain of Lypd6 terminated at Asn82, which is shorter than the ws-Lypd6 construct used for electrophysiology (terminated at Ala95). As, there are no experimental evidences that the *N*- and *C*-terminal regions do not participate in the interaction with nAChRs, the reliability of the obtained models may depend on the currently unknown function of these flexible tails.

**FIGURE 8:**
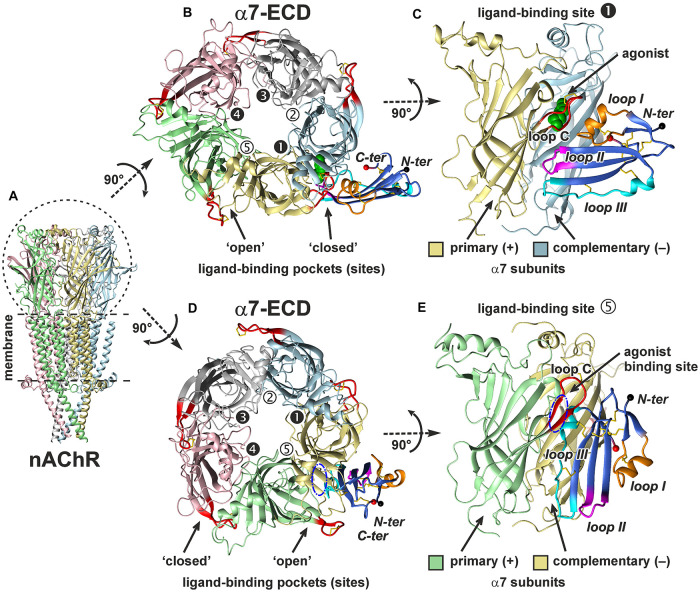
*In silico* modeling of the α7-ECD/ws-Lypd6 complex. **(A)** General view of homopentameric nAChR in the membrane. For clarity, the subunits are colored differently. **(B,D)** The top view on α7-ECD with the bound ws-Lypd6 molecules. Ligand-binding sites of the receptor have different conformations of the loop C. In the sites 1, 3, and 4 the ligand-binding pockets are “closed” by the loop C, while in the sites 2 and 5 the ligand-binding pockets are “open”. **(C,E)** The side view on the ligand-binding sites 1 and 5 of α7-ECD. Each binding site is formed at the interface between the primary (+) and complementary (–) α7 subunits. **(B,C)** Representative solution, where ws-Lypd6 contacts the “closed” α7-nAChR binding site with the loops I, II and III (complex #1, Supplementary Table 1). For clarity, the agonist (epibatidine) molecule (green spheres) is placed into the orthosteric ligand-binding site according to PDB id 3SQ6. **(D,E)** Representative solution, where ws-Lypd6 interacts with the “open” α7-nAChR binding site by the “head” and loop III (complex #2, Supplementary Table 1). The putative position of an agonist in the orthosteric binding site is shown by blue dashed-dot ellipses. C loop of the primary α7 subunit and ws-Lypd6 loops are shown by red and orange/magenta/blue colors, respectively. Disulfide bonds are in yellow. *N*- and *C*-termini of the Lypd6 LU-domain are shown with black and red spheres, respectively. The “favorable” contacts observed in the complexes are listed in Supplementary Table 1.

To account for a structural flexibility of the ws-Lypd6 LU-domain and α7-ECD, the combined molecular dynamics (MD)/ensemble protein–protein docking protocol was used (see section “Materials and Methods” and Supplementary Figure 2). MD simulation of ws-Lypd6 in the explicit water box revealed high-amplitude fluctuations in the loop regions of the protein, which agrees well with the previously obtained NMR data (Supplementary Figure 3; Paramonov et al., 2020). Similarly, the MD simulation of α7-ECD revealed the high conformational variability of the loop C (Figure 8, red). Some ligand-binding pockets were “open,” where the loop C of the primary α7 subunit protrudes into the solvent (Figures 8B,D, interfaces 2, 5), while the other sites were “closed” (the loop C is clamped to the receptor, Figures 8B,D, interfaces 1, 3, 4).

Obtained models of the α7-ECD/ws-Lypd6 complex are shown in Figure 8. The first model captures the interaction of ws-Lypd6 with the “closed” ligand-binding site of α7-ECD (Figures 8B,C). In this model the α-helix from the loop I of ws-Lypd6 (predicted binding site #1) interacts with the outer side of the receptor’s loop C forming multiple hydrogen bonds and ionic contacts. In addition, the loops II and III form contacts with the primary and complementary receptor subunits, respectively (Supplementary Table 1). Interestingly, the proposed Lypd6 binding site does not overlap with the agonist binding site (Figures 8B,C, green spheres). This agrees with the allosteric mode of the ws-Lypd6 action and with the observed absence of the competition with ACh.

The second model (Figures 8D,E) revealed the possible interaction of the “head” and loop III of ws-Lypd6 (predicted binding site #2) with the “open” ligand-binding pocket of α7-ECD. In this model the α-helix from the loop III of ws-Lypd6 penetrates the vestibule of the receptor’s ligand-binding site located under the loop C. The complex is stabilized by the contacts between the protein “head” and loop C of the primary subunit, while the loop III of the protein mainly interacts with the complementary subunit (Supplementary Table 1). However, in this model, the proposed Lypd6 binding site partially overlaps with the agonist binding site (Figures 8D,E, blue ellipse). In addition, in the obtained complex the tips of the loop regions are directed toward the membrane surface surrounding the receptor and could contact it (Figure 8E). Therefore, this mode of the interaction between α7-nAChR and Lypd6 was considered unlikely and is not discussed further.

## Discussion

Many receptors in the central nervous system have associated regulatory proteins expressed in the specific neurons (Maher et al., 2017). The auxiliary regulatory proteins have been described for the AMPA, NMDA, GABA_*A*_ receptors, and nAChRs. For example, the GPI-anchored Ly-6/uPAR protein Lynx1 is colocalized with α7-nAChRs in the brain areas important for learning and memory (Ibañez-Tallon et al., 2002), and is crucial for the loss of neuronal plasticity during postnatal development (Morishita et al., 2010) and regulation of the spine turnover in the adult visual cortex (Sajo et al., 2016). Lypd6 is also GPI-anchored Ly-6/uPAR protein expressed in the brain and, similarly to Lynx1, we observed its colocalization with α7-nAChRs in the cortical and hippocampal neurons (Figure 3). In addition, here, for the first time, we observed the colocalization of the Ly-6/uPAR protein with α3β4-nAChRs (Figure 4), which indicates that this receptor subtype may also possess auxiliary regulatory proteins. Thus, Lypd6 also could play some role in the regulation of the cholinergic signaling in the brain and cognitive processes.

*Lypd6* overexpression leads to increase the nicotine-evoked Ca^2+^-currents at heteromeric nAChRs in the trigeminal ganglia neurons in mice (Darvas et al., 2009). At the same time, knockout of the *Lypd6* gene also increases the nicotine-evoked currents in the dorsal raphe nuclei in transgenic mice (Arvaniti et al., 2018). Here, to resolve this controversy about the positive or negative modulatory role of Lypd6 in the cholinergic signaling, we studied the pharmacology of Lypd6 on a set of heteromeric and homomeric nAChRs using the isolated water-soluble LU-domain of the protein. We showed for the first time that ws-Lypd6 affects only α3β4- and α7-nAChRs (Figures 1A, 2A), demonstrating in the both cases the significant inhibitory activity. The incomplete inhibition observed at the α3β4 and α7 receptors (Figures 1B, 2B) and the absence of the competition with ACh (Figure 1C) suggest the allosteric modulatory mechanism of the ws-Lypd6 action. Given that the trigeminal ganglia neurons express different nAChR subunits including α7, α3, and β4 (Liu et al., 1998), we hypothesize that the previously described potentiation of heteromeric nAChRs (Darvas et al., 2009) was not due to the direct interaction of Lypd6 with the α3β4 receptors, but may be the result of the changes in the expression of heteromeric nAChRs conditioned by the Lypd6 overexpression. The connection of the nAChRs modulation in the neurons with the expression of nAChRs and other Ly-6/uPAR proteins was recently described (Bychkov et al., 2018).

The ability to inhibit several nAChR subtypes was shown previously for the other Ly-6/uPAR proteins. For example, Lynx1 and SLURP-2 are able to modulate not only the α7 receptors, but also α3β2-, α4β2-, and, probably, α3β4-nAChRs (Ibañez-Tallon et al., 2002; Lyukmanova et al., 2011, 2016b). At the same time, the α7 receptors are more abundant in the brain (Taly et al., 2009) and participate in the different cognitive processes including the regulation of the synaptic plasticity (Lozada et al., 2012; Koukouli and Maskos, 2015; Bali et al., 2017; Nicholson and Kullmann, 2021). Therefore, we focused our further work on the investigation of the Lypd6 interaction with α7-nAChRs. Previously we demonstrated that the water-soluble domain of human Lynx1 (ws-Lynx1; Lyukmanova et al., 2011) enhances the amplitude of the ACh-evoked currents at the α7 receptors in cortical slices (Shenkarev et al., 2020). In contrast, here we showed that ws-Lypd6 inhibits the choline-evoked currents at α7-nAChRs in hippocampal slices (Figure 5). This agrees well with the previously observed inhibition of the nicotine-evoked currents by GST-Lypd6 in hippocampal slices (Arvaniti et al., 2016).

Cholinergic inputs in the hippocampus are involved in the modulation of the synaptic plasticity underlying cognitive processes including learning and memory (Drever et al., 2011). Activation of nAChRs by nicotine results in the stimulation of LTP in the hippocampus (Welsby et al., 2009), while α-Bgtx and β-amyloid peptide (1–42) decrease LTP (Kapay et al., 2013; Bychkov et al., 2018; Richter et al., 2018). Here, we demonstrated that ws-Lypd6, similarly to α-Bgtx, significantly decreases LTP in the CA1 field of the hippocampus (Figure 6). The similarity of the effects observed upon the α-Bgtx and ws-Lypd6 treatment (Figure 6) together with the inhibition of the choline-evoked currents at α7-nAChRs in hippocampal slices (Figure 5) permit us to hypothesize, that the suppression of LTP by ws-Lypd6 is related to the negative modulation of α7-nAChRs. The “opposite” (relative to ws-Lypd6 and α-Bgtx) pharmacological properties of ws-Lynx1 at α7-nAChRs also support this hypothesis. Indeed, ws-Lynx1 is able to potentiate the ACh-evoked currents at the α7 receptors in the brain and significantly enhances LTP in the CA1 field of the hippocampus (Shenkarev et al., 2020).

The present and previous studies (Lyukmanova et al., 2011; Shenkarev et al., 2020) showed that the endogenous GPI-anchored neuromodulators (Lypd6, Lynx1, etc.) have a weak (tens of μM) affinity for nAChRs. This raises the question about the physiological significance of these interactions. A possible answer to this question is that these proteins should not regulate the receptors in the on-off fashion as neurotoxins do, which have the nanomolar affinity. Most likely, such proteins as Lypd6, Lynx1, and Lynx2 are required for the fine tuning of the nAChR function and/or for the regulation of the receptor maturation in the endoplasmic reticulum (Nichols et al., 2014) and/or for the control of the receptor expression at the cell surface (Wu et al., 2015). Moreover, the anchoring in the membrane near the receptor could increase the effective EC_50_ values of the modulatory Ly-6/uPAR proteins. The observed colocalization of Lynx1 and Lypd6 with nAChRs (Ibañez-Tallon et al., 2002; Figures 3, 4) supports this proposal.

**FIGURE 9:**
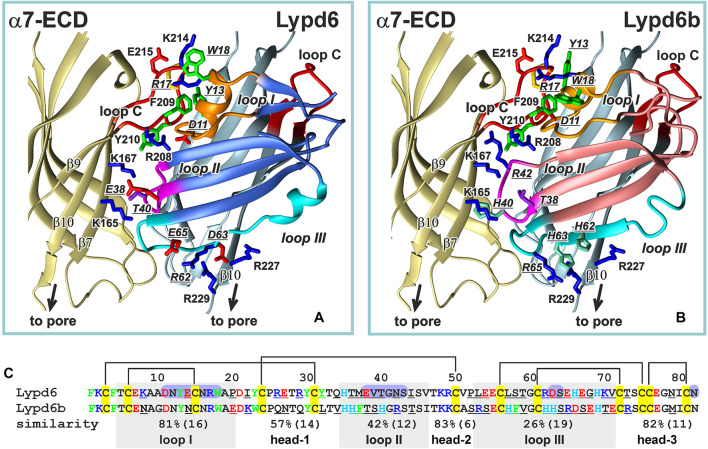
Comparison of the ws-Lypd6 and ws-Lypd6b amino acid sequences and their possible complexes with α7-ECD. **(A)** The “favorable” contacts in the α7-ECD/ws-Lypd6 complex (Figure 8C). The sidechains participating in the electrostatic and hydrophobic interactions are shown. **(B)** The possible structure of the α7-ECD/ws-Lypd6b complex. The ws-Lypd6b molecule is shown in the same orientation as ws-Lypd6 molecule in (A). Ws-Lypd6b cannot form as many “favorable” contacts with the receptor as ws-Lypd6. **(C)** Sequence alignment of the LU-domains of ws-Lypd6 and ws-Lypd6b. The similarity (%) is independently shown for each region of the proteins. The total number of residues in the loops is given in brackets. The differing residues are underlined. The aromatic (Phe, Trp, Tyr), positively charged (Arg and Lys), negatively charged (Asp and Glu), and His residues are colored in green, blue, red, and cyan, respectively. The residues of ws-Lypd6 forming contacts with α7-ECD in the proposed model are highlighted by violet background.

By analogy with the “three-finger” snake inhibitors of nAChRs,—α-Bgtx and α-Cbtx, we modeled the α7-ECD/ws-Lypd6 complex assuming that the Ly-6/uPAR protein binds near the orthosteric ligand-binding site located in the cleft between the primary (+) and the complementary (−) receptor’s subunits (Huang et al., 2013; Noviello et al., 2021). The orthosteric site is covered by the loop C of the primary subunit, which has high plasticity and can adopt several conformations. The “open” position of the loop C is usually observed in the complexes with the antagonists (e.g., α-Bgtx, conotoxin ImI) and corresponds to the closed channel pore, while the “closed” ligand-binding pocket was found in the complexes with the agonists (e.g., epibatidine, lobeline, nicotine) (Hansen et al., 2005). The movement of the loop C (its closure) triggers the conformational changes that lead to the opening of the channel pore (Law et al., 2005; Gulsevin, 2020; Noviello et al., 2021). The obtained docking solution (Figures 8B,C) indicates that the loops I, II, and III of ws-Lypd6 may interact with α7-nAChRs at the entrance to the orthosteric ligand-binding pocket. As shown in Figure 9A, in this case the loop I of the neuromodulator makes multiple contacts with the outer side of the loop C of the primary (+) receptor’s subunit in the “closed” position, while the loop III is involved in the electrostatic interactions with the β10-strand of the complementary (–) receptor’s subunit. The importance of these interactions is highlighted by the fact that the β10-strand connects the loop C of the nAChR subunit with the pore domain, and the β10-strand is required to translate the agonist-induced movement of the loop C into the pore opening. Thus, the predicted mode of the Lypd6 binding must hamper the nAChR channel activation that agrees with the inhibitory activity observed for ws-Lypd6 (Figures 1, 2, 5). Note, that in the electrophysiology experiments performed here, simultaneous responses from thousands of the channels were recorded. The observed decrease in the total current amplitude is probably caused by a decrease in the number of the open channels and not by a change in the conductivity of the individual channels.

According to our model, the Lypd6 binding site does not overlap with the agonist binding site at α7-nAChR (Figures 8B,C). This agrees with the proposed allosteric mode of the ws-Lypd6 action and with the observed absence of the competition with ACh (Figure 1C). It is interesting to compare the obtained α7-ECD/ws-Lypd6 model with the model proposed for the other allosteric modulator, e.g., Lynx1 (Lyukmanova et al., 2013). In the both cases the loop I of the Ly-6/uPAR proteins contacts the clamped loop C of the primary (+) α7 subunit, at the same time the positions of the loops II and III are different. In contrast to the situation observed for Lypd6, the positively charged residues of the Lynx1 loop II repel the positively charged residues of the β10-strand of the complementary (−) receptor’s subunit. Thus, contrary to ws-Lypd6, the bound Lynx1 molecule could promote the β10-strand movement and opening of the receptor pore, that may explain the opposite effects of ws-Lynx1 and ws-Lypd6 at α7-nAChRs.

In spite of the general similarity of the ws-Lypd6 and α-Bgtx effects at α7-nAChRs and LTP, the proposed mode of the human neuromodulator interaction with the α7 receptors differs significantly from the interaction mode of the competitive “three-finger” inhibitors, such as the snake α-neurotoxin α-Bgtx (Huang et al., 2013; Noviello et al., 2021). In contrast to Lypd6, which binds at the entrance to the orthosteric site, the neurotoxin’s loop II contacts the inner side of the loop C of the primary (+) receptor’s subunit and occupies the site that is usually occupied by the agonists, making impossible the receptor activation in the presence of α-Bgtx. Interestingly, the tip of the toxin’s loop II contains the pair of the positively charged and aromatic residues (Arg36 and Phe32), which are crucial for the high affinity binding of α-neurotoxins with the nicotinic receptor (Antil-Delbeke et al., 2000). Toxin’s loop II interacts with the Tyr210 residue from the inner side of the loop C (Noviello et al., 2021), while the loop I of the Lypd6 molecule also contains the pair of positively charged and aromatic residues (Tyr13 and Arg17), but in our model these residues interact with the aromatic moiety of the Phe209 residue located in the outer side of the loop C (Figure 9A). Lypd6 and α-Bgtx are relatively large molecules, thus their simultaneous interaction with the loop C, although with its different parts, is sterically impossible. Thus, the proposed model of the α7-ECD/Lypd6 complex explains both the allosteric mode of the neuromodulator action and its competition with α-Bgtx (Arvaniti et al., 2016).

Among the human Ly-6/uPAR proteins, Lypd6b has the highest amino acid sequence similarity with Lypd6 (∼54%, Figure 9C and Supplementary Figure 1). It was reported previously, that Lypd6b negatively modulates α3β4-nAChRs and has no activity against the α7 receptors (Ochoa et al., 2016). Here, using the isolated water-soluble LU-domain of Lypd6b (ws-Lypd6b), we observed the weak inhibitory activity of ws-Lypd6b at α7-nAChRs expressed in *X. laevis* oocytes, but the magnitude of the current suppression was much lower than that of ws-Lypd6 (Figures 2B,C). Usually, the loops of the Ly-6/uPAR proteins are the main epitopes for interaction with their targets (Vasilyeva et al., 2017). Comparison of the Lypd6 and Lypd6b sequences revealed ∼81, 42, and 26% of similarity of the loops I, II, and III, respectively (Figure 9C). These differences are translated to the different surface properties of the proteins (Figures 7B,D) and could lead to the lower stability of the α7-ECD/ws-Lypd6b complex. Indeed, despite the high contain of the identical residues in the loop I (Supplementary Table 1), the Lypd6 residues in the loops II and III forming the favorable contacts with the receptor are not conserved in Lypd6b (Figure 9C). For example, the fragments Glu38-Val-Thr40 (loop II) and Arg62-Asp-Ser-Glu65 (loop III) of Lypd6 form the electrostatic contacts with the β7(+)- and β10(−)-strands of the receptor and correspond to the fragments Thr38-Ser-His40 (loop II) and His62-His-Ser-Arg65 (loop III) of Lypd6b, which have the different charge distribution (Figures 9A,B). Thus, the proposed mode of the Lypd6 interaction with α7-nAChR can also explain the weaker inhibitory activity of Lypd6b.

*D.rerio* Lypd6 participates in the formation of the Wnt/β-catenin receptor complex, which is involved in the embryo development (Özhan et al., 2013). The interaction of the LU-domain of human Lypd6 with the Wnt coreceptor LRP6 was described recently (Zhao et al., 2018). Here we describe the alternative targets of Lypd6,—α3β4- and α7-nAChRs. The IC_50_ value of ws-Lypd6 at α7-nAChRs expressed in *Xenopus* oocytes (∼10 μM, Figure 2C) is close to Kd of the Lypd6/LRP6 complex (∼1 μM, Zhao et al., 2018). Moreover, 1 μM ws-Lypd6 robustly inhibits α7-nAChR (by 28%) in hippocampal slices (Figure 5C). Thus, Lypd6 is the multi-targeting protein, and besides enhancing of the Wnt-dependent signaling (Özhan et al., 2013) it can also down-regulate α7-nAChRs. Notably, the ability to interact with the receptors from the structurally different families has been shown for several Ly-6/uPAR proteins. For example, the snake neurotoxin WTX and human proteins ws-Lynx1 and SLURP-2 are able to interact with nAChRs and the muscarinic receptors (Lyukmanova et al., 2011, 2015, 2016b), while other snake neurotoxin α-cobratoxin can inhibit both nAChRs and the GABA_*A*_ receptors (Kudryavtsev et al., 2015). Modeling of the α7-nAChR/Lypd6 complexes (Figure 8) indicates that the simultaneous Lypd6 interaction with LPR6 and α7-nAChR is sterically hindered. It is likely that the interaction with one of these receptors scavenges the GPI-tethered Lypd6 molecule and prevents its binding to another target. In this case, the long and flexible 19-residue linker between the LU-domain and the GPI-anchor is needed to adapt the position of the LU-domain to the binding sites on the different receptors. For comparison, the site of the GPI-anchor attachment in the Lynx1 molecule is located directly at the *C*-terminus of the LU-domain, while in CD59 and Lynx2 the linkers are seven and nine residues long, respectively (Supplementary Figure 1).

In summary, we described for the first time the pharmacology of the human neuromodulator Lypd6 at nAChRs. The findings suggest that Lypd6 is the negative modulator of α3β4- and α7-nAChRs in the brain. At the same time, positive modulation of the α7 receptors by the other neuromodulator Lynx1 from the Ly-6/uPAR family was described previously (Ibañez-Tallon et al., 2002; Lyukmanova et al., 2011; Shenkarev et al., 2020). It is likely that Lynx1 and Lypd6, both co-expressed with α7-nAChRs on the neuronal membranes, represent the pair of the positive and negative modulators, required for the fine-tuning of the cholinergic signaling in the brain. The positive and negative effects on the synaptic plasticity (as illustrated by increase or decrease in LTP, see Shenkarev et al., 2020; Figure 6) imply that Lynx1 and Lypd6 can also differently modulate the processes involved in cognition, memory, and learning. Comparison of the possible modes of the interaction of Lynx1 and Lypd6 with the extracellular ligand-binding domain of α7-nAChR revealed that the positive and negative modulation of the receptor can be realized through the different, but partially overlapping binding sites.

## Data Availability Statement

The original contributions presented in the study are included in the article/Supplementary Material, further inquiries can be directed to the corresponding author/s.

## Ethics Statement

The animal study was reviewed and approved by the Ethical Committee of the Shemyakin-Ovchinnikov Institute of Bioorganic Chemistry RAS for the control of the maintenance and use of animals (protocol #222 from 13 February 2018).

## Author Contributions

AC, DK, SKoz, ELy, IS, and ZS participated to research design. MB, AC, SKos, DK, ELo, AP, IP, MS, and SP conducted the experiments. JT, RE, MK, SKoz, ELy, and VS contributed to new reagents and analytic tools. SKos, DK, ELy, AP, and ZS performed the data analysis. RE, SKoz, DK, IP, IS, ZS, VS, and ELy wrote and contributed to the writing of the manuscript. All authors reviewed the manuscript.

## Conflict of Interest

The authors declare that the research was conducted in the absence of any commercial or financial relationships that could be construed as a potential conflict of interest.

## Publisher’s Note

All claims expressed in this article are solely those of the authors and do not necessarily represent those of their affiliated organizations, or those of the publisher, the editors and the reviewers. Any product that may be evaluated in this article, or claim that may be made by its manufacturer, is not guaranteed or endorsed by the publisher.

## Supplementary Material

Supplementary Figure 1 Amino acid sequence alignment of Ly-6/uPAR proteins of different origin. The signal peptides are removed. Invariant Cys residues are shown in yellow. Cys residues forming additional disulfide bonds in loop regions are shown in green. Disulfide bonds are shown by brackets. Predicted sites for attachment of a GPI-anchor and glycosylation are shown by cyan and magenta, respectively. Several possible GPI-anchor sites were predicted for human Lypd6b. Fragments of the Lypd6 and Lypd6b proteins used in this work are highlighted by orange background. The *N*- and *C*-terminal boundaries of the LU-domain are shown by vertical dashed lines. ^∗^The proteins for which the presence of both the GPI-anchored and soluble form is reported. The loop regions are highlighted with a gray background.

Supplementary Figure 2 Customized MD/ensemble protein–protein docking protocol and a two-step post-scoring procedure to select the most probable α7-ECD/ws-Lypd6 complex.

Supplementary Figure 3 Conformational plasticity of ws-Lypd6. Comparison of root-mean-square fluctuation (RMSF) in MD trajectories of ws-Lypd6 with NMR generalized order parameters (S^2^, mean ± SD), which characterize the amplitude of the backbone motions at the ps-ns timescale. RMSF values are shown for six 200 ns trajectories [three trajectories for ws-Lypd6 and three trajectories for ws-Lypd6 with removed *C*-tail (Leu83-Ala95)]. Threshold value (S^2^ < 0.8) distinguishes residues with high-amplitude motions at ps-ns timescale. The regions of the loops and *C*-tail are highlighted by a gray background. Two short loops in the “head” region are denoted as “head-1” and “head-2.” The S^2^ values are taken from Paramonov et al. (2020).
