# Supplementary figures and images for: Human Three-Finger Protein Lypd6 Is a Negative Modulator of the Cholinergic System in the Brain
Source: Front Cell Dev Biol. 2021 Sep 21;9:662227. doi: 10.3389/fcell.2021.662227 (PMC8494132; doi:10.3389/fcell.2021.662227)

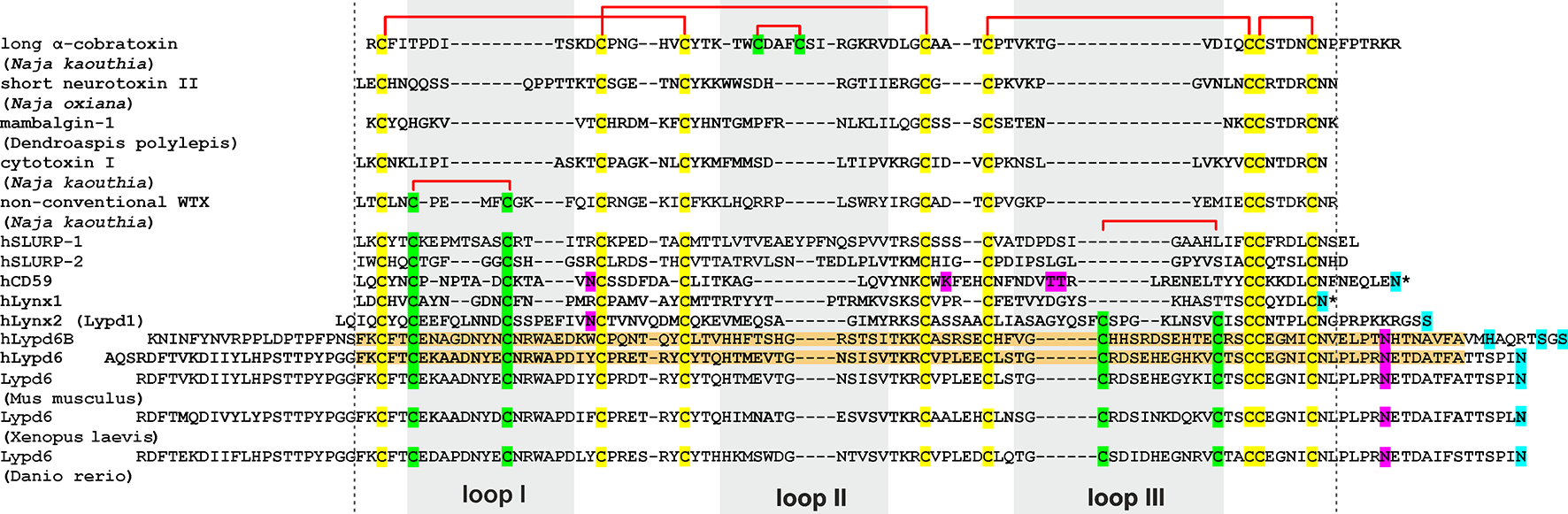

Supplement: Supplementary Figure 1 — Amino acid sequence alignment of Ly-6/uPAR proteins of different origin. The signal peptides are removed. Invariant Cys residues are shown in yellow. Cys residues forming additional disulfide bonds in loop regions are shown in green. Disulfide bonds are shown by brackets. Predicted sites for attachment of a GPI-anchor and glycosylation are shown by cyan and magenta, respectively. Several possible GPI-anchor sites were predicted for human Lypd6b. Fragments of the Lypd6 and Lypd6b proteins used in this work are highlighted by orange background. The N- and C-terminal boundaries of the LU-domain are shown by vertical dashed lines. ∗The proteins for which the presence of both the GPI-anchored and soluble form is reported. The loop regions are highlighted with a gray background. [file Image_1.tif]

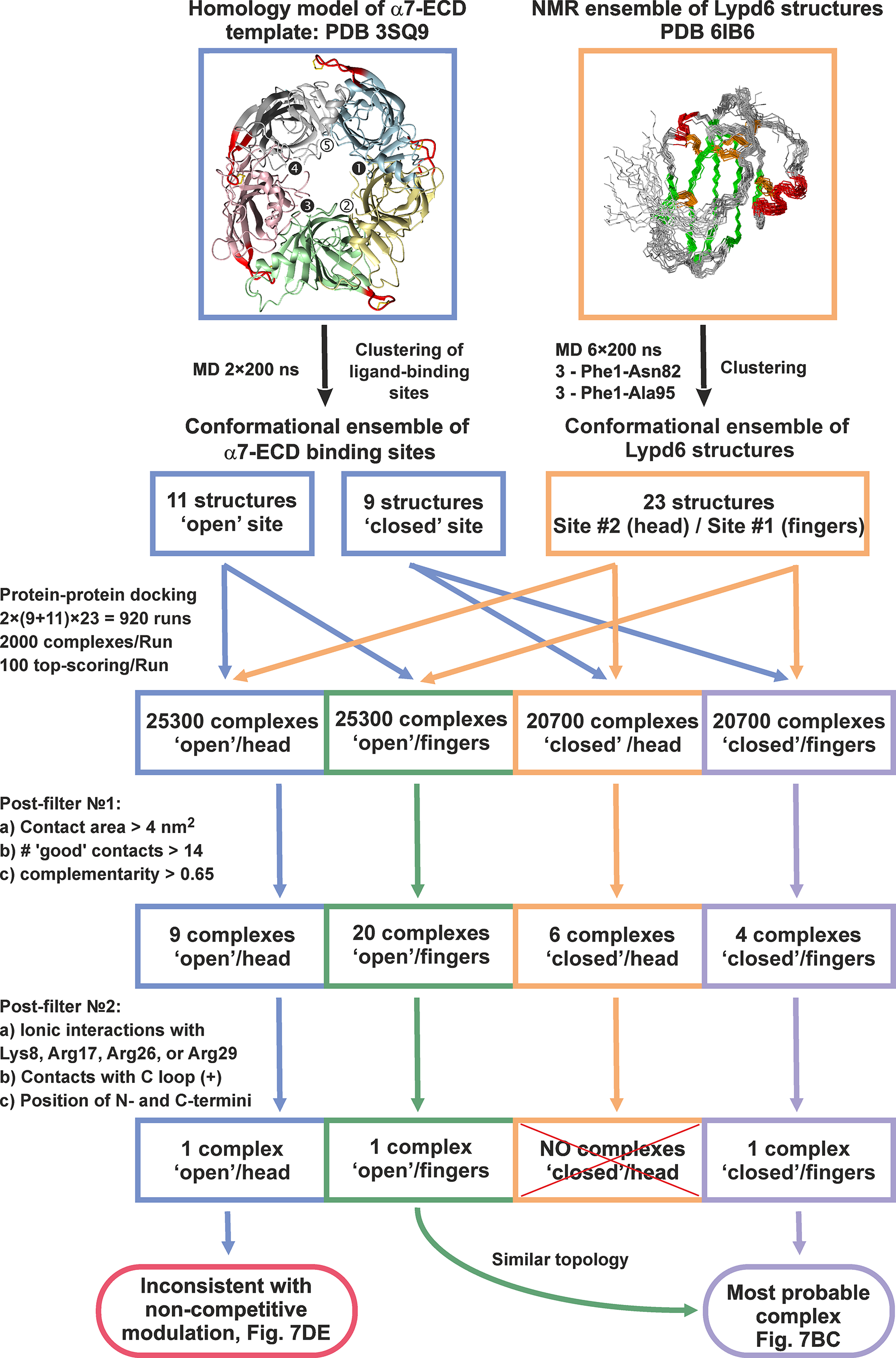

Supplement: Supplementary Figure 2 — Customized MD/ensemble protein–protein docking protocol and a two-step post-scoring procedure to select the most probable α7-ECD/ws-Lypd6 complex. [file Image_2.tif]

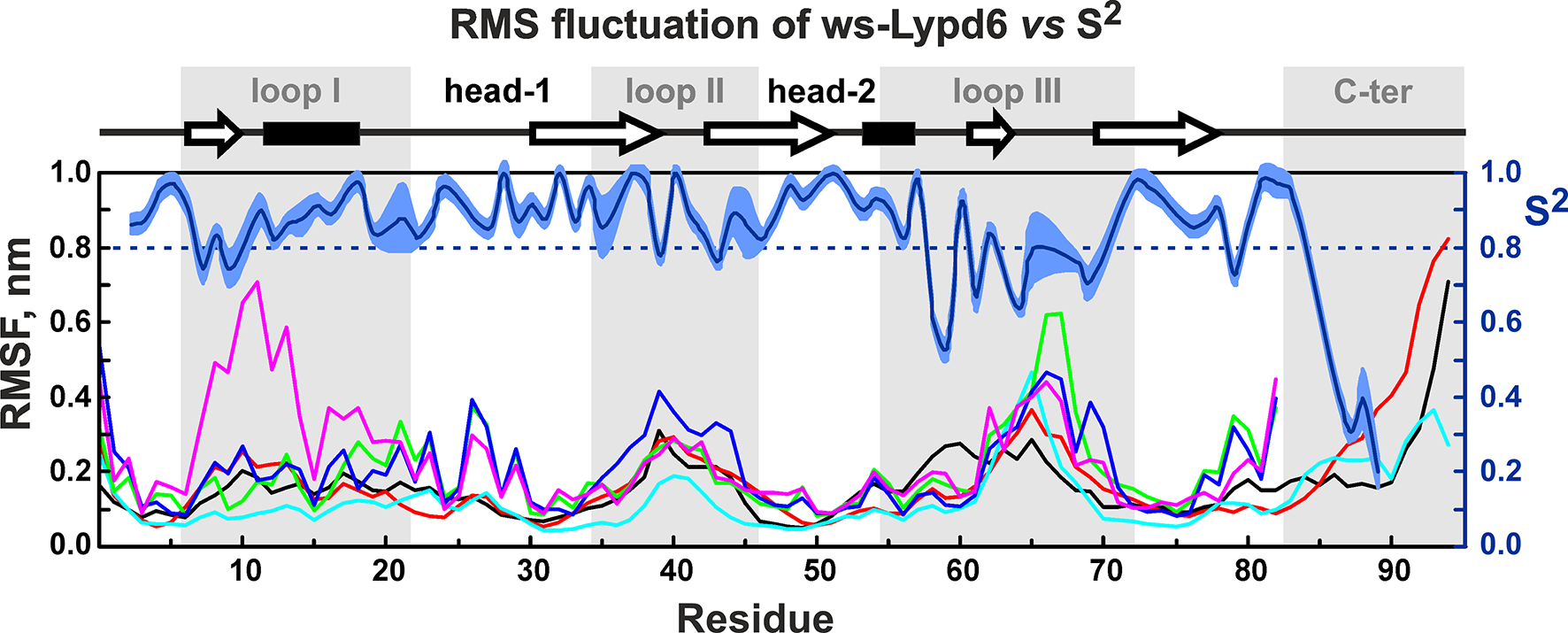

Supplement: Supplementary Figure 3 — Conformational plasticity of ws-Lypd6. Comparison of root-mean-square fluctuation (RMSF) in MD trajectories of ws-Lypd6 with NMR generalized order parameters (S2, mean ± SD), which characterize the amplitude of the backbone motions at the ps-ns timescale. RMSF values are shown for six 200 ns trajectories [three trajectories for ws-Lypd6 and three trajectories for ws-Lypd6 with removed C-tail (Leu83-Ala95)]. Threshold value (S2 < 0.8) distinguishes residues with high-amplitude motions at ps-ns timescale. The regions of the loops and C-tail are highlighted by a gray background. Two short loops in the “head” region are denoted as “head-1” and “head-2.” The S2 values are taken from Paramonov et al. (2020). [file Image_3.tif]
