# Supplementary material for: Human Three-Finger Protein Lypd6 Is a Negative Modulator of the Cholinergic System in the Brain
Source: Front Cell Dev Biol. 2021 Sep 21;9:662227. doi: 10.3389/fcell.2021.662227 (PMC8494132; doi:10.3389/fcell.2021.662227)
Supplement: Supplementary file 4 [file Table_1.docx]

**Table 1S. Contacts between ws-Lypd6 and α7-ECD in the modeled complexes.**

| **Lypd6 residue** | **Lypd6b residue^a^** | **Complex #1**  **(loops I, II and III)** | | **Complex #2**  **(‘head’ and loop III)** | |  |
| --- | --- | --- | --- | --- | --- | --- |
|  |  | **α7-ECD, residue** | **α7-ECD, loop** | **α7-ECD, residue** | **α7-ECD, loop** |  |
| **loop I** | | | | | | |
| E7 | E |  |  | K204 (I) | β9(-) |  |
| D11 | D | R208 (I) | loop C(+) |  |  |  |
| Y13 | Y | F209 (S,M); C212 (M); C213 (H,M); Q139 (H) | loop C(+); loop C(+); loop E(-) |  |  |  |
| R17 | Y | F209 (S); E215 (I); C213 (H) | loop C(+) |  |  |  |
| W18 | W | K214 (H) | loop C(+) |  |  |  |
| **‘head-1’** | | | | | | |
| R26 | ***Q**** |  |  | S135 (H); H137 (S) | loop E(-) |  |
| R29 | ***Q**** |  |  | E211 (I); E215 (I,H) | loop C(+) |  |
| Y30 | Y |  |  | Y210 (M) | loop C(+) |  |
| **loop II** | | | | | | |
| E38 | ***T**** | K165 (I); K167 (I,H) | β7(+) |  |  |  |
| N42 | ***R**** | S206 (H); Y210 (H) | loop C(+) |  |  |  |
| S43 | S | R208 (H) | loop C(+) |  |  |  |
| **‘head-2’** | | | | | | |
| R49 | K |  |  | D186 (I,H) | loop F(-) |  |
| L53 | ***R**** |  |  | Y217 (M) | loop C(+) |  |
| **loop III** | | | | | | |
| L57 | ***H**** |  |  | Y115 (H,M);  W171 (M) | loop A(+);  loop B(+) |  |
| R62 | ***H**** |  |  | G194 (H); D197 (I) R229 (H,S) | loop F(-); β10(-) |  |
| D63 | ***H**** | R227 (I); R229 (I) | β10(-) | K165 (I) | Β7(+) |  |
| S64 | S | D197 (H); R229 (H) | loop F(-); β10(-) |  |  |  |
| E65 | ***R**** |  |  | R229 (I) | β10(-) |  |
| E67 | ***S**** |  |  | R229 (I) | β10(-) |  |
| H69 | H |  |  | R227 (S) | β10(-) |  |
| V71 | ***E**** |  |  | L198(M); V199(M) | loop F(-) |  |
| T73 | ***R**** |  |  | I187 (M) | loop F(-) |  |
| ***C*-ter** | | | | | | |
| E77 | E |  |  | R208 (I) | loop C(+) |  |
| N82 | N | E184 (H) | loop F(-) |  |  |  |

H, S, I, and M in brackets denote the types of interaction: hydrogen bond, stacking, ionic bond, and hydrophobic contact (according to molecular hydrophobicity potential), respectively. (+) and (-) denote primary and complementary subunits of the receptor, respectively. The loops forming orthosteric ligand-binding site of nAChR are given according to (Grutter and Changeux, 2001). The modeled complexes are shown in Figure 7.

^a^) Residue of Lypd6b in homologues position. Non homologues replacements are shown by italic-bold and marked with asterisks.
